# Supplementary material for: Epigenetic Reprogramming and Inheritance of the Cellular Differentiation Status Following Transient Expression of a Nonfunctional Dominant-Negative Retinoblastoma Mutant in Murine Mesenchymal Stem Cells
Source: Int J Mol Sci. 2024 Oct 3;25(19):10678. doi: 10.3390/ijms251910678 (PMC11476944; doi:10.3390/ijms251910678)

Supplementary figures

Sequencing of DM miniprepped plasmid DNA used for gene promoter methylation analysis. Primers used to amplify fragments are highlighted in blue. Detected CpGs are highlighted in yellow.

Supplementary Figure S 1. Representative chromatogram of the *Cebpa* promoter showing methylation of 37 CpGs detected in the *Cebpa* promoter of undifferentiated ΔS/N cells.

File: E01_E01_05_RapidSeq50_POP7_Z.ab1 Run Ended: May 18, 2021, 11:18:38 Signal G:697 A:613 T:874 C:314 Comment:
Sample: E01 Lane: 5 Base spacing 11.54 679 bases in 8099 scans

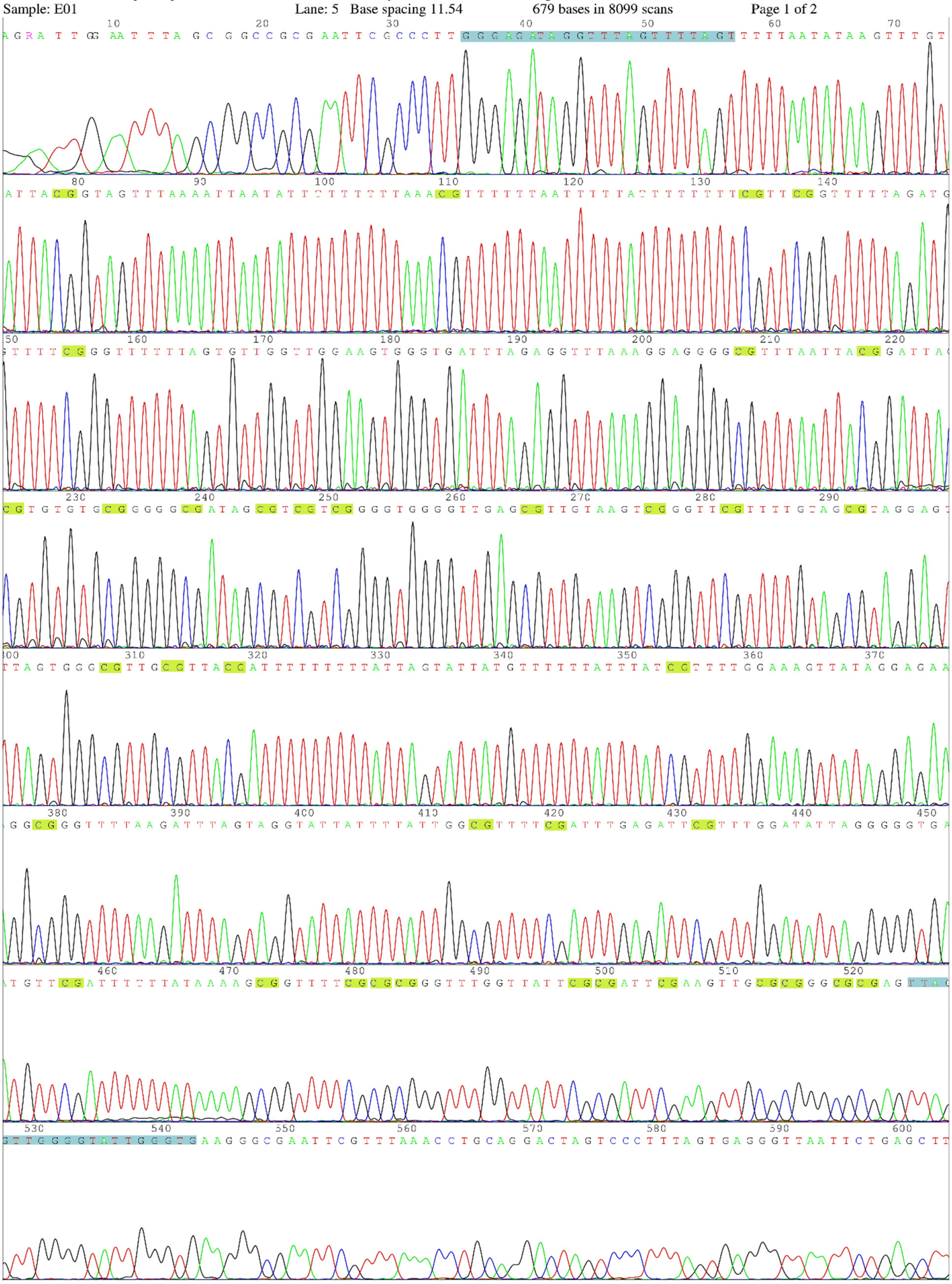

Supplementary Figure S 2. Representative chromatogram of the *Cebpa* promoter showing CpG methylation detected in the Cebpa promoter of differentiated ΔS/N cells.

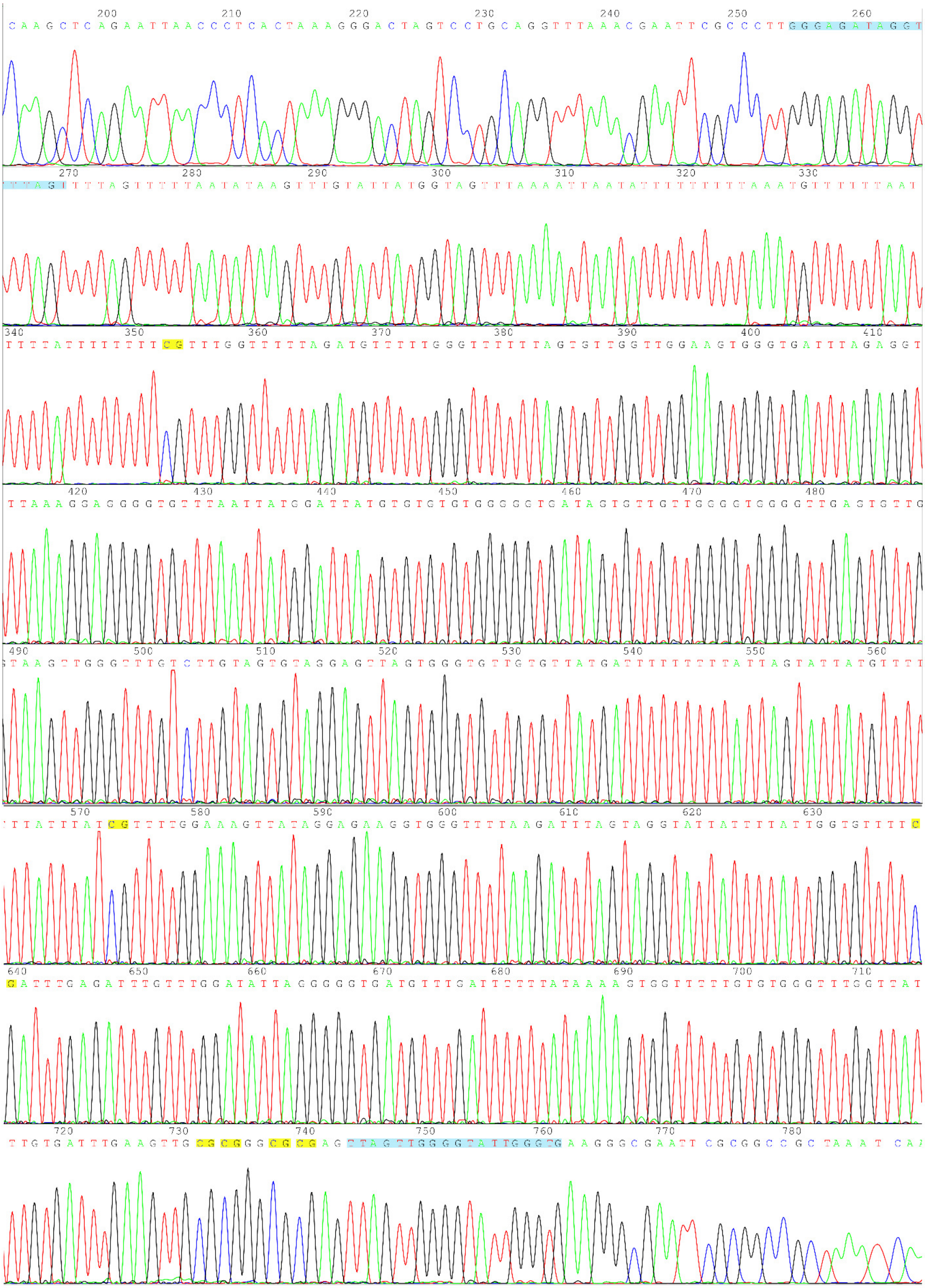

Supplementary Figure S 3. Representative chromatogram of the 722 bp *Ezh2* promoter showing CpG methylation detected in differentiated ΔS/N cells.

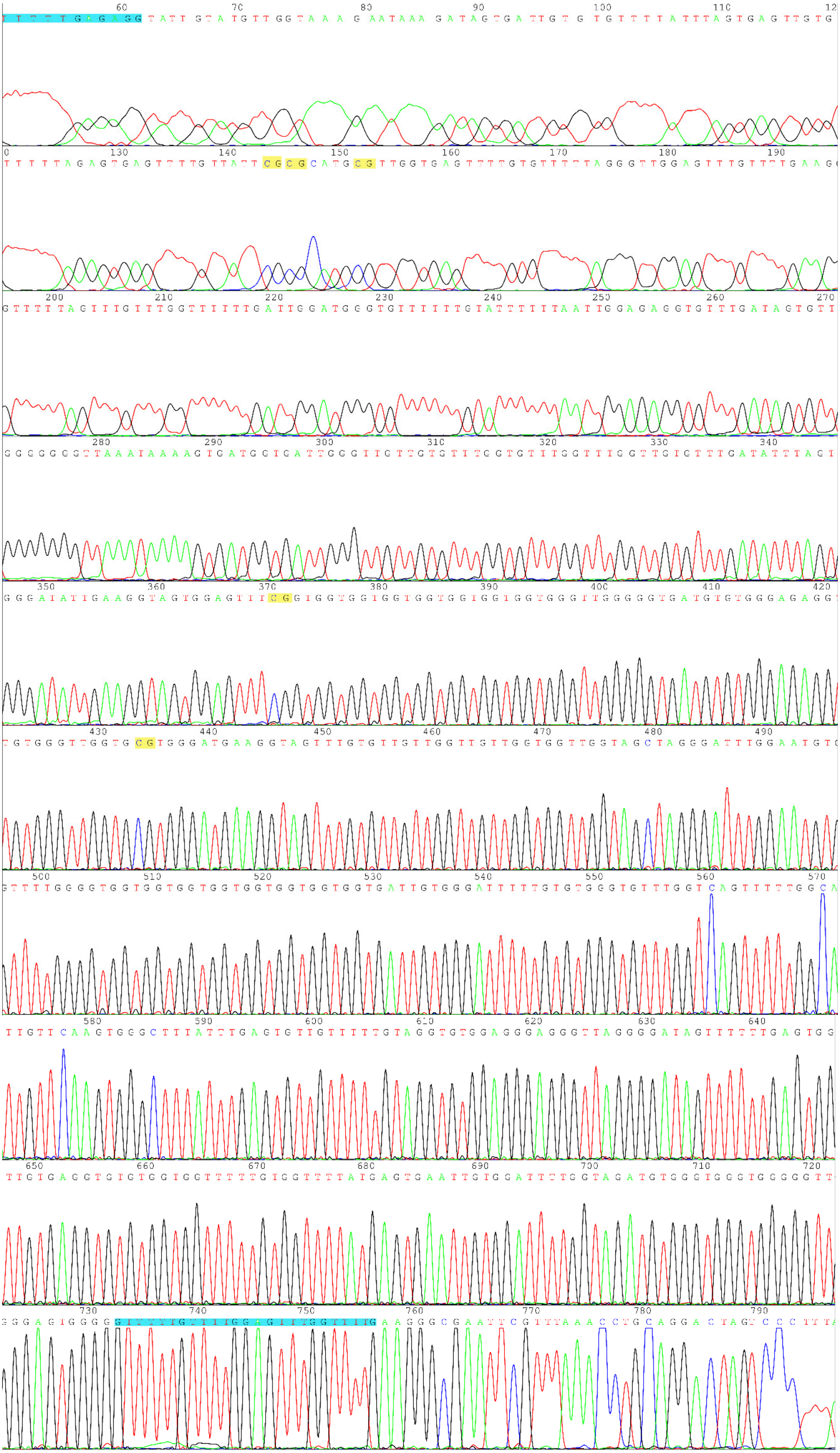

Supplementary Figure S 4. Representative chromatogram of the 696 bp *Mll4* promoter showing CpG methylation detected in differentiated ΔS/N cells.

Sample: F12 Lane: 6 Base spacing 10.80 701 bases in 8688 scans Page 1 of 2

10 20 30 40 50 60

T T T A G G G G G G T G T T G T T G A T T A T A G G T G G T T A G G T T G G G G G T G G G G T A G T T T T T G A G G T T A G A G

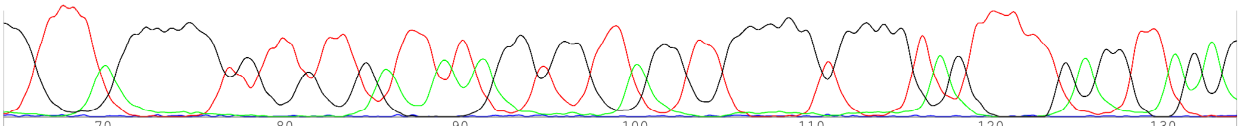

70 80 90 100 110 120 130

G G G G A A G G G A G G T G A G T G T T G G G T T T G G A G T G G T T G G A G G G G A G T G G G T A G A G G G T T T G T A T T G T T T G T T T

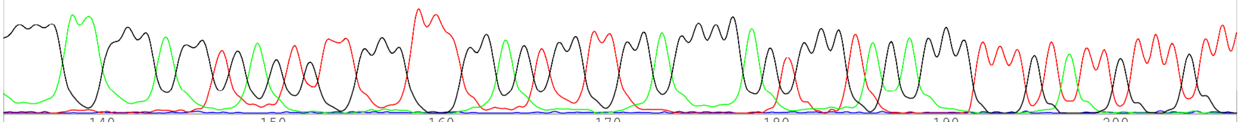

140 150 160 170 180 190 200

T T T T T T T T T T T T T G T T T A T T A A T T T T T T T T T T T T T T T T G G G G G A G T A G A A G G T G G G G G T T T G A A G T T G T T G

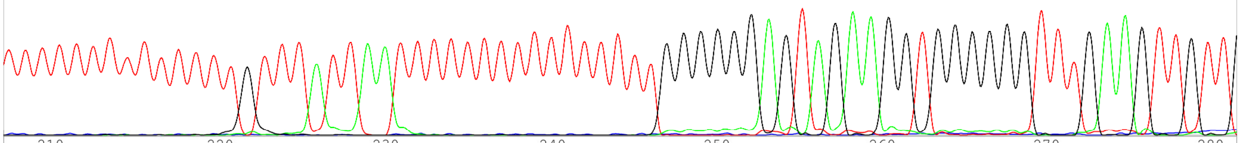

210 220 230 240 250 260 270 280

G A G G G G G A C G T T T G G G G T T G T G G A G T T T G G T G T T G G G T G T G T G T T A G G T T T G G T T T T T G T T G G T T T T G T G T T G T T

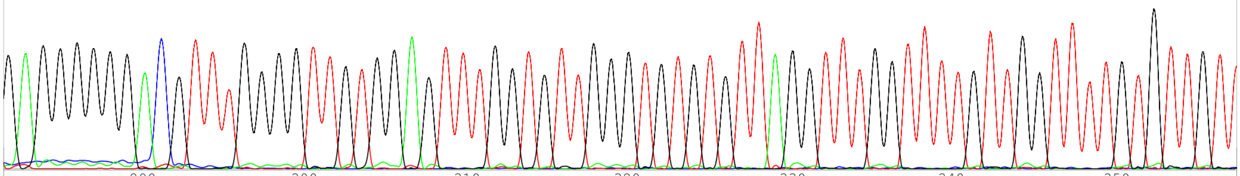

290 300 310 320 330 340 350

T G T A G T T T G T G T G G T T T T G G G G T G T T G G T T T G G T G G G G A G A G G G G T T T G G T G T G G T T G T G A G G G G T T T A T G T T T T A T

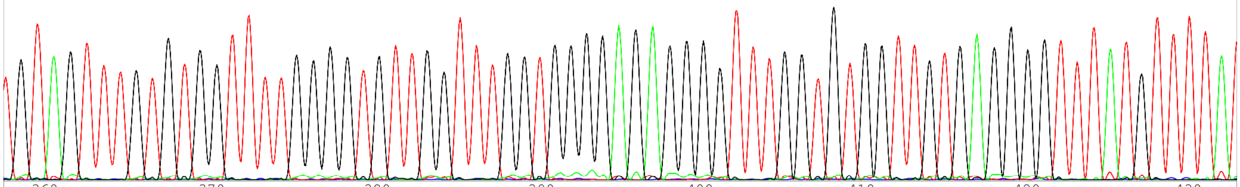

360 370 380 390 400 410 420 430

T T T G G G T T T G G A G T G T G G T G T G G T G T G G T G T G G T A T T T T T T T T T G G G T T G T T G G G G A G G T G T T T T G A T G T T T T A T T T

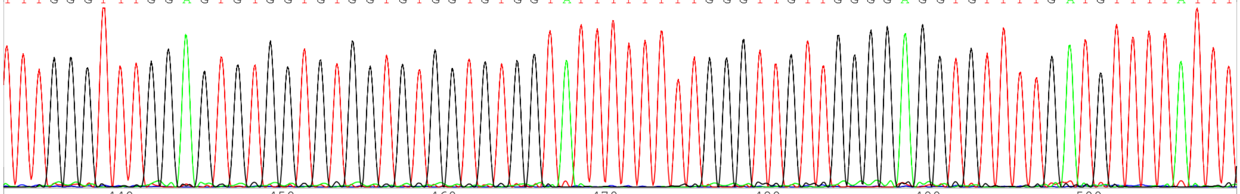

440 450 460 470 480 490 500

G G A G A G T T T T G A T G T T T T G T T T T G A G T T T G G T G T G G G T G G T T G G G G T G T T G G T T G G G T T T T A G G A T T G A G A G G T C G T

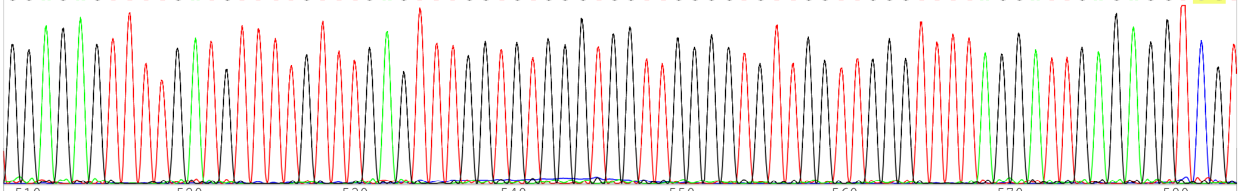

510 520 530 540 550 560 570 580

T T T T G T G A T G T G G A T G T G G A G T T T T G T T T G T T G T T T A A G A T T A A A G T T A T T G G T G T T T T T T T A G T G T T T G T T T G G G A T T T

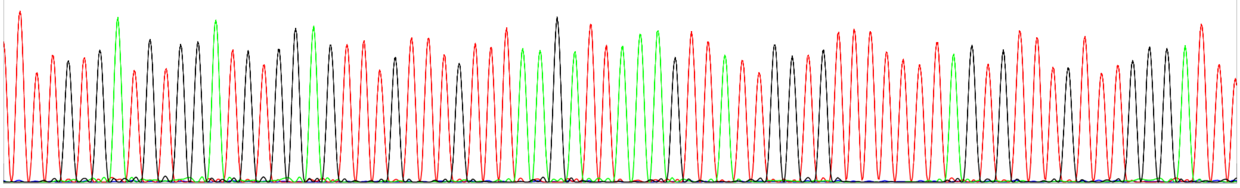

File: F12_F12_06_RapidSeq50_POP7_Z.ab1 Run Ended: Nov 12, 2021, 22:06:17 Signal C:151 T:2635 A:1769 G:3226 Comment:

Sample: F12 Lane: 6 Base spacing 10.80 701 bases in 8688 scans Page 2 of 2

590 600 610 620 630 640 650

T G T T G T T T T G G G G T T G T T T A T G G A A A T T T A A A T T G T T G G A A T T T G A G G T A G A G A A T T T T T G T T G G A A G G G C G A A T

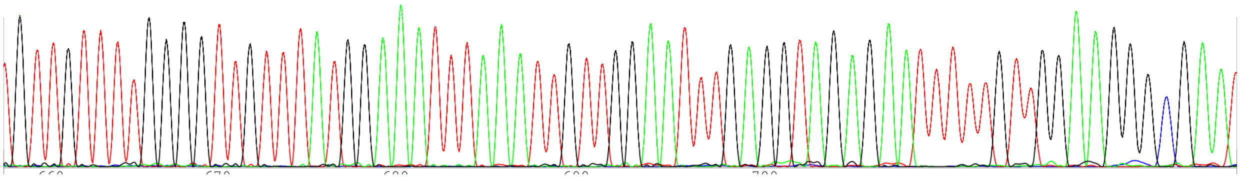

Supplement: Supplementary file 1 [file ijms-25-10678-s001.zip › ijms-3212043-supplementary.pdf]
